# Marginal adaptation of bulk-fill resin composites with different viscosities in class II restorations: a micro-CT evaluation

**DOI:** 10.1186/s12903-024-03975-7

**Published:** 2024-02-13

**Authors:** İsmail Hakkı Baltacioğlu, Gülbike Demirel, Burcu Öztürk, Fulya Aydin, Kaan Orhan

**Affiliations:** 1 Department of Restorative Dentistry, Faculty of Dentistry, Ankara University, Ankara, Turkey; 2 Department of Restorative Dentistry, Faculty of Dentistry, Ankara Medipol University, Ankara, Turkey; 3 Department of Maxillofacial Radiology, Faculty of Dentistry, Ankara University, Ankara, Turkey

**Keywords:** Bulk fill composites, Marginal adaptation, Micro-ct

## Abstract

**Background:**

The purpose of this study is to evaluate the marginal adaptation of bulk-fill resin composites with different viscosities (paste-like and flowable) in Class II restorations using micro-CT imaging.

**Methods:**

Forty extracted human molars were used. Mesial and distal Class II box cavities (approximately 3 mm x 3 mm x 4 mm) were prepared for each tooth, with cavity floors located 1 mm below the enamel-cementum junction. Following adhesive application, teeth were restored using eight different groups: Group XB: X-tra Base Bulk-fill Flowable (VOCO), Group XF: X-tra Fill Bulk-fill (VOCO), Group FB: Filtek Bulk-fill Posterior (3 M ESPE), Group FF: Filtek Bulk-fill Flowable (3 M ESPE), Group BB: Beautifil-Bulk (SHOFU), Group BF: Beautifil-Bulk Flowable (SHOFU), and Group CO: “as a control group”, Clearfil Majesty Posterior (KURARAY) and Group CF: “as a control group”, Clearfil Majesty Flow + Clearfil Majesty Posterior (KURARAY). The restored teeth underwent an aging protocol involving 1000 cycles in a water bath fluctuating between 5 ± 1.0 °C and 55 ± 1.0 °C. Post-aging, teeth were immersed in 50% silver nitrate solution for 24 h and then in a film developer solution for 8 h. Microleakage analysis was performed using micro-CT, evaluated with 3D Slicer software. A two-way ANOVA was employed for statistical analysis.

**Results:**

Two-way ANOVA results indicated significant effects of both viscosity (*p* < 0.0001) and composite type (*p* < 0.0001) on marginal adaptation. Viscosity analysis (comparing flowable and paste-like) revealed no significant differences in the FB-FF, XB-XF and BB-BF groups but significant differences in the and CO-CF group, with flowable type exhibiting less microleakage than paste-like type.

**Conclusions:**

The study suggests that while the viscosity of bulk-fill composites did not significantly affect marginal adaptation, the brand of bulk-fill composite did influence it.

## Background

Composite resin materials are widely utilized in dental restorations due to their excellent esthetic properties and favorable mechanical characteristics [1–3]. Achieving optimal marginal adaptation, which refers to the precise fit and integration of the composite restoration with the tooth structure at the restoration margins, is crucial for the long-term success and durability of the restoration [3]. However, the conventional incremental layering technique used in composite restorations has certain limitations. One of the primary disadvantages is the time-consuming nature of the technique. Layering each increment individually requires meticulous attention and can extend the chairside time for the clinician. Moreover, there is a risk of failure in interlayer bonding due to potential contamination between layers, which can compromise the overall integrity of the restoration [4, 5]. Additionally, the incremental technique may result in the formation of voids or gaps between the layers, which can contribute to microleakage and secondary caries formation [4–7]. To address these limitations, bulk-fill composites have been developed as an alternative approach to simplify the restoration procedure and overcome the drawbacks of the incremental technique [8–10]. With bulk-fill composites, thicker increments can be placed, reducing the need for multiple layers and potentially saving clinical time. The bulk-fill technique minimizes the risk of contamination between increments and by reducing the number of layers, the potential for void formation is also reduced [8, 11].

Bulk-fill composites are available in different viscosities, typically categorized as “flowable” or “paste-like.” The viscosity of the composite material affects its handling properties and clinical applications. Flowable bulk-fill composites have a lower filler content, which gives them a more fluid consistency [12, 13]. This flowability allows for easier adaptation into cavities and can simplify the placement process. However, flowable bulk-fill composites generally exhibit lower wear resistance compared to conventional composites due to their reduced filler content [8, 14–16] To overcome this limitation, it is often recommended to cap the top layer of flowable bulk-fill composites with a conventional composite that has a higher filler content [17]. On the other hand, paste-like bulk-fill composites have a higher filler content, which imparts increased viscosity, sculptability, and wear resistance [8] These composites are less flowable compared to their flowable counterparts but still offer the advantage of bulk placement in thicker increments. Due to their higher filler content, paste-like bulk-fill composites generally do not require capping with additional composite layers for enhanced wear resistance [13, 18].

While numerous studies have investigated the marginal adaptation of bulk-fill composites in general [11–13, 18–20], there is a gap in the literature specifically addressing the impact of viscosity variations within the bulk-fill composite category. Understanding the influence of different viscosities on marginal adaptation is crucial for selecting the most suitable bulk-fill composite material for specific clinical scenarios. Therefore, the aims of this study were to compare the marginal adaptation of bulk-fill composites with different viscosities using Micro-CT imaging. The null hypotheses tested in this study as follows: (1) The difference in viscosity between flowable and paste-like bulk-fill composites would not have a significant effect on marginal adaptation. (2) The chemistry differences between the composites would not affect the marginal adaptation.

## Methods

This study was conducted under all the provisions of the World Medical Association Declaration of Helsinki and the Ankara University Faculty of Dentistry’s local human subjects oversight committee guidelines and policies of the ethics committee for the study of humans and animals (No:36,290,600/12/2022).

### Tooth selection and specimen preparation

**Fig. 1:**
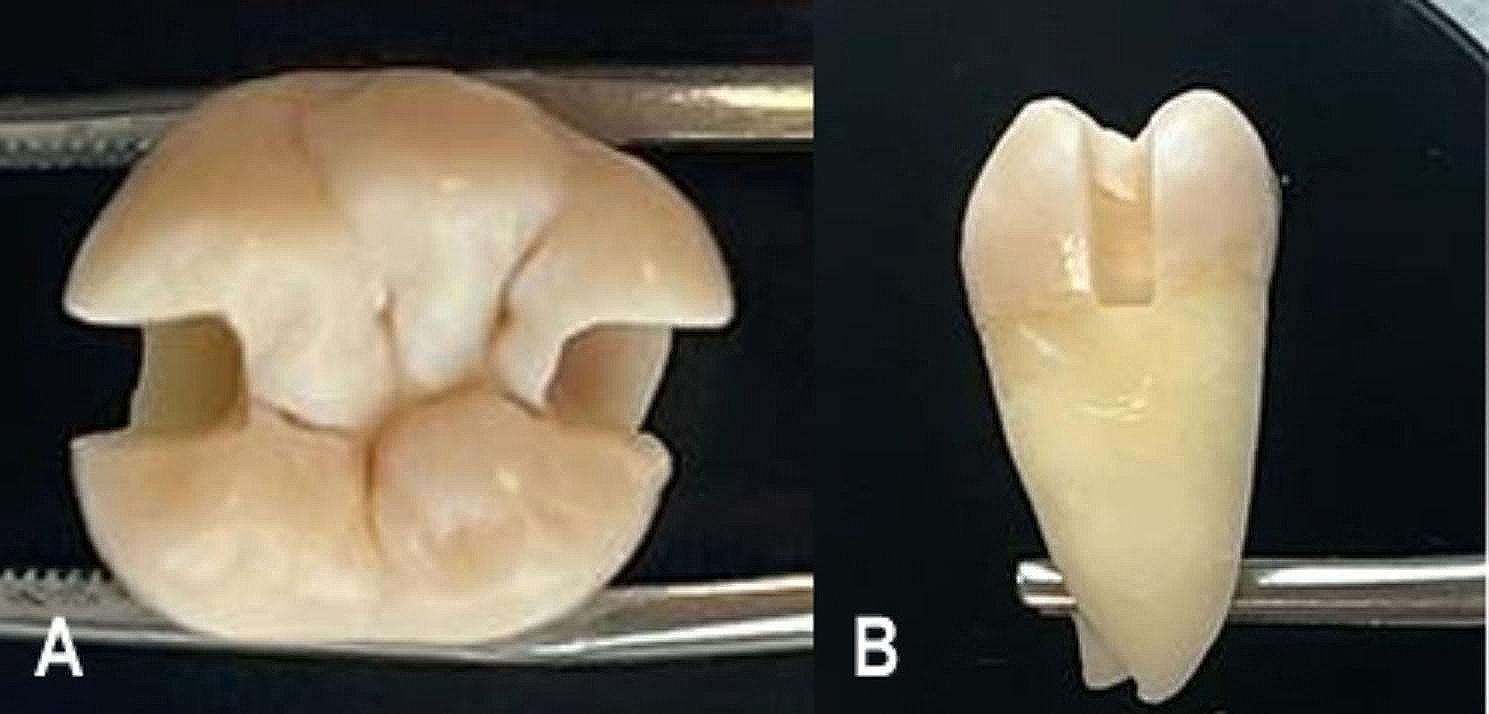
Standardized Class II cavity preparation. **A**: Occlusal view **B**: Aproximal view

Many previous microleakage studies have employed a sample size of minimum 8–10 [21–23]. In parallel with these studies, this research has also arranged the sample size to be 10 per group. A total of forty caries-free human molars were selected and teeth were stored in distilled water at a temperature of 25ºC to maintain dentin permeability until sample preparation. The teeth were then randomly divided into eight groups, with each group consisting of five teeth (*n* = 5). Standardized box-shaped Class II cavities were prepared mesial and distal to each tooth and the cavities were prepared 1 mm below the cervical border of the enamel-cementum junction (Fig. 1). Mesial and distal class II box cavities with similar dimensions (≈ 3mmx3mmx4mm) were prepared for each tooth with a rounded-ended cylindrical diamond bur, using a high-speed handpiece under air-water spray. The cavities were standardized to a depth of 4 mm by abrading the occlusal surfaces. Cavity dimensions were measured and checked with a periodontal probe, and teeth that did not meet these standards were eliminated. Buccal and lingual walls of the cavity were shaped parallel to each other, and the cave surface margins lacked a bevel. Burs were changed after every five uses.

### Restorative procedures

Restorative Materials Used in the Study were shown in Table 1. After cavity preparation, Clearfil S^3^ Bond Plus was applied to all cavity walls using a single-use brush in a brushing motion for 10s. Subsequently, the solvent was evaporated with a gentle air stream for 5s. This was followed by polymerization for 10s using a LED curing device (Radii plus, SDI Limited, Australia). Before each sample, it was ensured that the light intensity was higher than 1300mW/cm² with a radiometer device. After the adhesive application, the following procedures were applied to the groups.

**Table 1:** Composition of restorative materials used in the study

Restorative Materials and their Abbreviations	Colour	Composition	Manufacturer	Lot number
X-tra base bulk fill flowable, (XB)	U	Bis-EMA, aluminum, and barium silicate Filler Content (wt%): 75 (average filler size: 1 μm)	VOCO, Cuxhaven, Germany	2,029,511
X-tra fill bulkfill, (XF)	U	Bis-GMA, TEGDMA, UDMA, barium aluminum silicate, fumed silica, and pigments Filler Content (wt%): 86 (average filler size: 2–3 μm)	VOCO, Cuxhaven, Germany	2,029,440
Beautifil Bulk Flowable, (BF)	U	F-Al-Si glass, Bis-GMA, Bis‐MPEPP, TMGDMA Filler Content (wt%): 73 (average S-PRG filler size: 0.8 μm)	SHOFU, Kyoto, Japan	22,041
Beautifil Bulk Restorative, (BB)	U	Bis-GMA, UDMA, Bis- MPEPP, TEGDMA, S-PRG filler based on F-Al-Si glass, polymerization initiator, pigments, others, Filler Content (wt%): 87 (average S-PRG filler size: 0.8 μm)	SHOFU, Kyoto, Japan	31,828
Filtek bulkfill flowable, (FF)	U	Silane treated ceramics, UDMA, BIS-EMA-6, YbF3, BISGMA, TEGDMA, ethyl 4-dimethylaminobenzoate Filler Content (wt%): 65 (filler size range of 0.01 to 5 μm)	3 M ESPE, USA	NC36728
Filtek bulkfill posterior, (FB)	A2	DMA, DDMA, AUDMA, Zirconia/silica cluster filler, ytterbium flüoride Filler Content (wt%): 77 (filler size range of 0.004-0.1 μm)	3 M ESPE, USA	N693117
Clearfil majesty flow, (CF)	A2	Hydrophobic aromatic, dimethacrylate, TEGDMA, Camphoroquinone, Barium glass filler, silica filler. Filler Content (wt%): 81 (filler size range 3–20 μm)	Kuraray Co. Ltd., Osaka, Japan	8A0322
Clearfil Majesty Posterior, (CO)	A2	Bis-GMA, hydrophobic aromatic, dimethacrylate, prepolymerized organic, filler, camphoroquinone, Silane barium glass. Filler Content (wt%): 92 (filler size range 1.5–20 μm)	Kuraray Co. Ltd., Osaka, Japan	370,001
Clearfil S3 Bond	-	MDP, HEMA, Bis-GMA, water, ethanol, photo-initiator, silanated colloidal silica	Kuraray Co. Ltd., Osaka, Japan	13

Abbreviations: **Bis-EMA**: Bisphenol A Ethoxylate Dimethacrylate, **Bis-GMA**: Bisphenol A Glycidyl Methacrylate, **TEGDMA**: Triethylene Glycol Dimethacrylate, **UDMA**: Urethane Dimethacrylate, **F-Al-Si glass**: Fluoride Aluminum Silicate Glass, **Bis-MPEPP**: Bisphenol A Methacrylate Phosphate Ester Prepolymer, **TMGDMA**: Tetramethylene Glycol Dimethacrylate, **S-PRG filler**: Surface Pre-Reacted Glass Ion Filler, **YbF3**: Ytterbium Fluoride, **DMA**: Dimethacrylate, **DDMA**: Dodecyl Methacrylate, **AUDMA**: Aliphatic Urethane Dimethacrylate, **BIS-EMA-6**: Ethoxylated Bisphenol A Dimethacrylate, **MDP**: 10-Methacryloyloxydecyl Dihydrogen Phosphate, **HEMA**: Hydroxyethyl Methacrylate

Restorative material was applied as a single layer of 4 mm and polymerized for 10 s to Group XB, XF, BB, FF. Restorative material was applied as a single layer of 4 mm and polymerized for 10s separately from the occlusal, buccal and lingual surfaces in Group FB. In control group CF; Clearfil Majesty flow was applied as a single layer of 2 mm and polymerized for 20s and then Clerfil Majesty Posterior was applied as two layer of 1 mm and each layers was polymerized for 20s. Clearfil Majesty Posterior was applied in 3 layers, two layers of 1.5 mm and a single layer of 1 mm, and each layer was polymerized for 20 s to other control group CO.

### Aging procedures and mCT analysis

After the restorative procedures, the samples were immersed in distilled water for 24 h at 37 °C prior to the thermal cycling process. Artificial aging was applied to the samples in a thermal cycle device (SD-Mechatronic, Westerham, Germany). As in other microleakage studies [24–26], the specimens were thermally aged for 1000 cycles in a water bath at 5 ± 1.0 °C and 55 ± 1.0 °C with a dwell time of 30s and 7s for transfer time. The specimens were kept in AgNO_3_ (Merckb 101,510 Silver Nitrate cryst, merck Kga, 64,271, Germany) solution prepared at a ratio of 1/1 in the dark at room temperature for 24 h. Silver-impregnated teeth were then rinsed with distilled water and kept in Dental X-ray Developer solution (Medley, MDC, Turkey) for 8 h under fluorescent light to reduce silver or diamine silver ions to metallic silver particles and rinsed with distilled water.

All samples were scanned using a micro-CT system (Bruker Sky- scan 1275, Kontich, Belgium). The teeth were positioned with the buccal surface facing the X-ray tube to ensure standardization. The scanning conditions were as follows: 100 kVp, 120 mA, 0.5-mm Al/Cu filter, 9.1-lm pixel size, and rotation at 0.1 steps. Other parameters, including ray hardening correction and optimum contrast, were adjusted according to the manufacturer’s instructions.

**Fig. 2:**
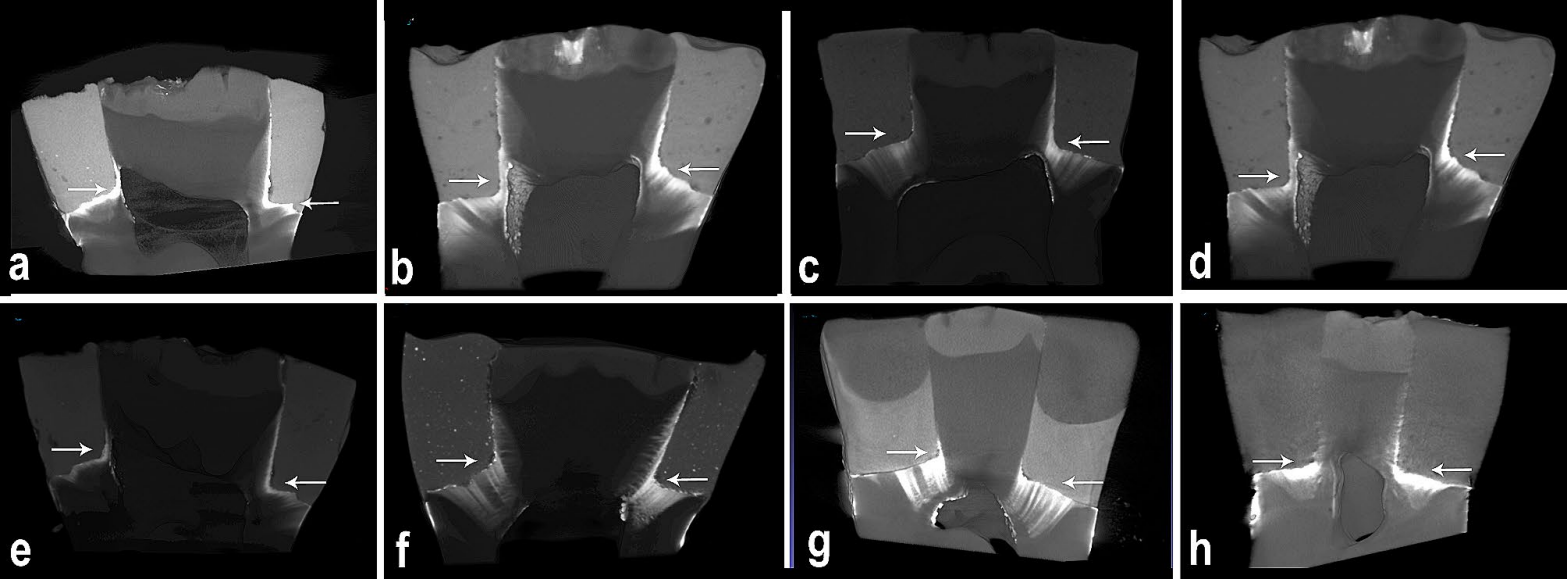
Representative sagittal micro-ct images. “white arrows” indicate AgNO_3_ penetrations. **a**: XF group. **b**: XB group. **c**: BF group. **d**: BB group. **e**: FB group. **f**: FF group **g**: CF group. **h**: CO group

**Fig. 3:**
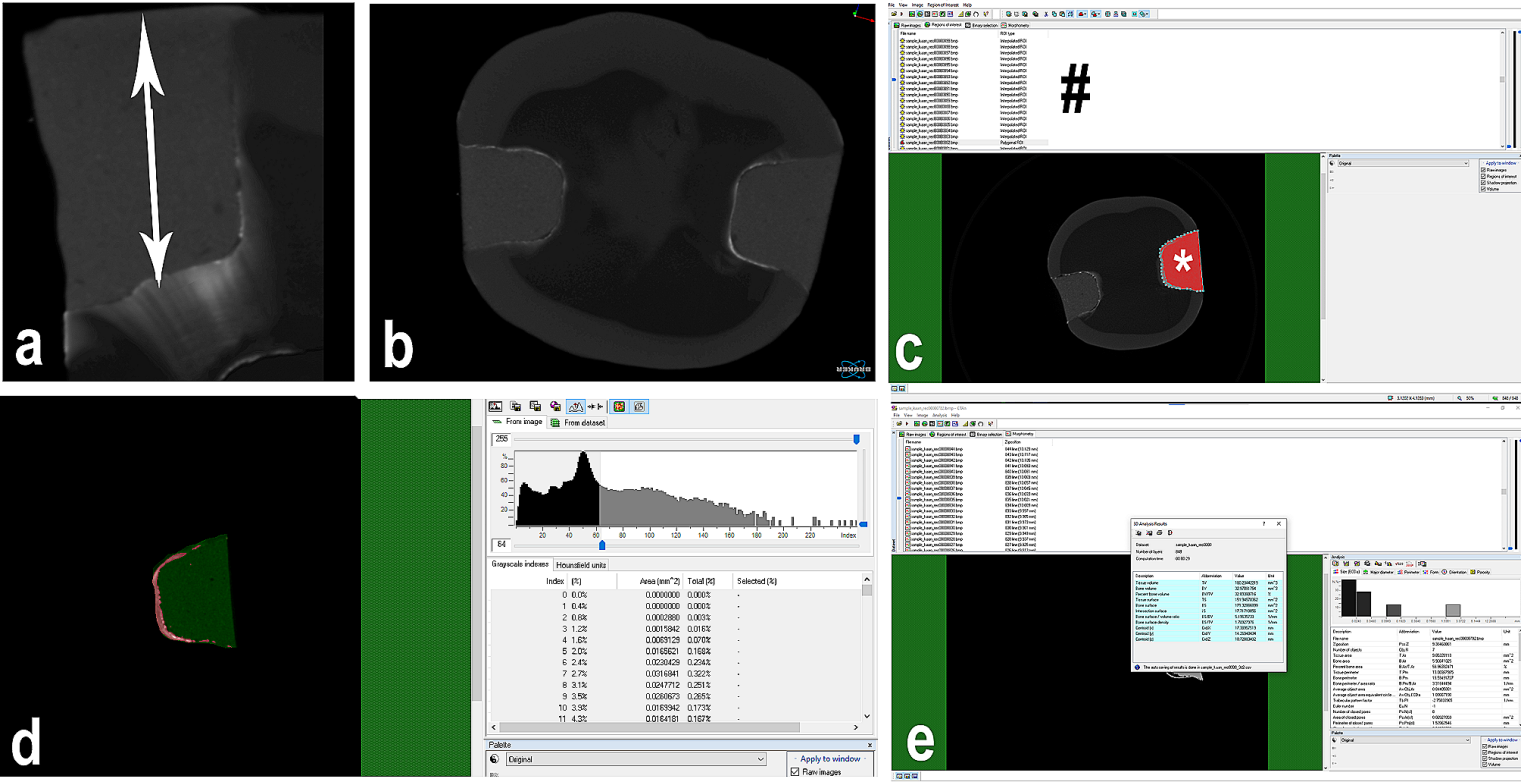
Measuring Processes. **a**: Determination of the restoration length in the sagittal plane. **b**: Axial view of a sample. **c**: Selection of sections to be used **#**; list of sections used, *; selected ROI. **d**: The parts outside the ROI was discarded. Segmentation of silver nitrate(red) and restoration(green) were carried out with different threshold values. **e**: Automatic calculation of the total amount of restoration and the total amount of silver nitrate in the ROI.

Representative images of AgNO_3_ filled in the gaps between the filling and tooth interface were shown in Fig. 2. CTAn program was used to calculate the volume of silver nitrate leaking from the restoration edges. A region of interest (ROI) was drawn to include the restoration and AgNO_3_ in the sample using CTAN software, and all features of the program were used to analyze the microarchitecture of each sample (Fig. 3). In the software, the total volume of silver nitrate and the total volume of restorations within the relevant ROI were calculated in “mm³”. These values were then divided to determine the leakage rates for the respective samples.

### Statistical analysis

The distribution of the data was evaluated using the Shapiro-Wilk normality test. Since a homogeneous distribution was achieved, a two-way analysis of variance (ANOVA) was employed as the statistical method. For multiple comparisons, Sidak test used for the viscosity effect, and the Tukey test was used for brand effect.

## Results

**Fig. 4:**
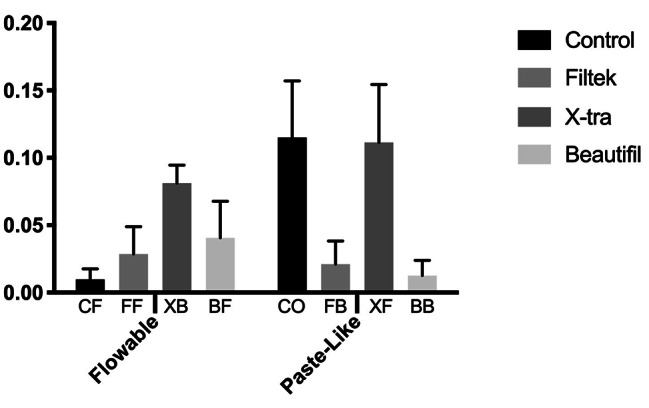
Mean leakage values and standard errors of the groups

Results of the ANOVA was shown in Table 2. Statistically significant differences found in terms of marginal adaptation for different composite brands, among groups with varying viscosities of the same brand and interaction. (*p* < 0.05, Fig. 4).

**Table 2:** Results of the 2-way ANOVA for silver-nitrate penetration ratios

2-way ANOVA	SS	DF	MS	F (DFn, DFd)	*P* value
Viscosity Factor	0,06908	3	0,02303	F (3, 72) = 33,91	*P* < 0,0001
Brand Factor	0,01253	1	0,01253	F (1, 72) = 18,45	*P* < 0,0001
Interaction	0,05174	3	0,01725	F (3, 72) = 25,40	*P* < 0,0001
Residual	0,04890	72	0,0006791		

Upon comparing composite groups with low viscosities (Table 3), the control group CF exhibited the lowest leakage, with no statistical difference from the FF group (*p* > 0.05). However, the BF and XB groups exhibited statistically higher leakage than the CF group (*p* < 0.05). While no difference was observed between FF and BF groups (*p* > 0.05), the XB group demonstrated significantly higher microleakage compared to all other groups (*p* < 0.05).

**Table 3:** Results of Tukey and Sidak multiple comparison tests for silver-nitrate penetration rates, mean and standard errors of restorative groups and their significant differences

Multiple Comparisons							
Flowable Viscosity				Paste-Like Viscosity			
Brand abb.	Mean and Std Err	Sig.*		Brand abb.	Mean and Std Err	Sig.*	
		Tukey	Sidak			Tukey	Sidak
**CF**	0,01 − 0,002	A	x	**CO**	0,12 − 0,013	B	y
**XB**	0,11 − 0,01	C	x	**XF**	0,08 − 0,004	B	x
**FF**	0,03 − 0,006	AB	x	**FB**	0,02 − 0,005	A	x
**BF**	0,04 − 0,008	B	x	**BB**	0,01 − 0,004	A	x

*Same lower-case letters in the same row and same upper-case letters in the same column have no significant difference

In the comparison of composite groups with paste viscosities (Table 2), the BB and FB groups displayed lower leakage values, differing significantly from the control group CO and XF (*p* < 0.05). No statistical distinction was found between CO and XF groups, as well as between BB and FB groups (*p* > 0.05).

Analyzing viscosity differences within the same brand of composites, the Filtek group (FF and FB), x-tra (XB and XF) and Beautifil group (BB and BF) showed no significant differences in leakage rates (*p* > 0.05), whereas distinctions were observed between the control group (CF and CO) (*p* < 0.05). Notably, in this group, composite with a paste consistency exhibited higher leakage.

## Discussion

One of the most important features expected from a dental restoration is maximum compatibility and adaptation to dental tissues [27]. However, applying such restorative materials in a challenging environment like the mouth poses difficulties for the dental practitioner [28]. Bulk-fill composites, which eliminate the need for incremental layering application inherent to traditional composites, are also expected to exhibit good marginal adaptation [12]. Marginal adaptation is influenced by various factors, including the viscosity and application method of the materials, their composition, polymerization shrinkage, and the stresses that may arise post-shrinkage [29–31]. In this context, the aim of this study was to evaluate the marginal adaptation of bulk-fill resin composites with different viscosities in Class II restorations using micro-CT.

The assessment of marginal adaptation was conducted through the quantification of leakage in three dimensions (3D), achieved using micro-computed tomography (micro-CT) as referenced in [32, 33]. Based on the idea that there is a correlation between the marginal adaptation properties of the material and cavity configuration [34], Class II composite cavities were strategically prepared in molars, allowing a more precise assessment of marginal adaptation properties. To simulate the demanding conditions prevalent in the oral environment, this study employed a thermal aging protocol after restorative procedure [35, 36].

Findings of this study suggest that the brand-specific properties, rather than the material viscosity, significantly influence the marginal adaptation of tested composites. Despite differences in viscosity, flowable and paste-like bulk-fill composites exhibited similar microleakage. However, in the control group, flowable composites adapted better to Class II cavity geometries than their paste-like counterparts, this could be due to their lower filler content and greater fluidity, which might aid in sealing marginal gaps [37–41]. Conversely, paste-like composites might generate more stress during polymerization, leading to increased leakage [39, 42, 43]. These observations led to the partial acceptance of our first null hypothesis, asserting that viscosity differences do not significantly affect marginal adaptation in bulk-fill composites. However, conventional control composites exhibited significant differences.

The rejection of the second null hypothesis was based on the observed impact of composite chemistry on marginal integrity. Specifically, the XB group (low viscosity) and XF group (paste viscosity) showed higher leakage compared to other bulk-fill and control groups, underscoring the influence of brand-specific properties. Similar to the present study, Nascimento et al. found that XB exhibited higher marginal leakage compared to other tested composites [44]. They speculated that the observed outcome could be attributed to the smaller filler size of XB. However, this explanation does not align with our study, as the sizes of the fillers in the composite groups we tested are similar. Despite this, the XB and XF groups have shown more leakage in the present study. Other studies examining the marginal adaptation of bulk-fill composites have demonstrated that, compared to other bulk-fill groups, the X-tra groups (XB and XF) exhibit lower marginal adaptation [9, 45].

According to the result of this study, when compared with control groups, it was observed that in terms of flowable viscosity, higher adaptation was exhibited by the CF and FF groups. Conversely, in paste viscosity, the low adaptation was shown by the CO and XF groups. The superior adaptation demonstrated by the CF group might be attributed to its chemical composition and the application technique of 1 mm layer thickness. The lower adaptation observed in the CO group might be due to its chemical composition, a high filler ratio (w %92), and its application method involving three separate increments, distinct from other groups. This similarity indicates that bulk-fill composites could be viable alternatives to traditional materials.

Studies present varied perspectives on the marginal adaptation of bulk-fill composites. While some authors [13, 41, 46] assert their superior marginal adaptation, others, reported different findings. Miletic et al. [47] showed that, with accept of Filtek Z250, conventional composites showed less gingival leakage than bulk-fill composites. Benetti et al. [9] found that no differences in bulk-fill composites with respect to conventional composites, but showed that larger gaps in two of the low-viscosity bulk-fill resins (XB and Venus Bulk Fill). Comparisons with conventional composites in various cavity types also yielded diverse results. Numerous Studies [48–51] found no significant difference in microleakage between bulk-fill and conventional composites in Class II restorations. However, Atmaja and Dewanto [52] noted less microleakage in Class V cavities with bulk-fill composites, indicating that cavity type might influence outcomes.

The current study results in emphasis on the marginal adaptation of different viscosities contrast with the conclusions of studies such as those by Miletic et al. [47] and Cayo-Rojas et al. [48], which did not find significant differences in performance based on viscosity. This situation may be due to the difference in the methods used for evaluating marginal adaptation in studies. Notably, both studies have investigated dye leakage by examining only one section with a microscope while the present study used Micro-ct and analyzed the whole regions of the specimen. Another possible reason is; the possibility that the impact of viscosity may be more material-specific than previously thought, further underscoring the importance of chemical composition.

However, this study has limitations. The different insertion techniques (bulk technique and incremental technique) may have influenced stress formation and, consequently, leakage. Additionally, the incremental technique increased polymerization steps and energy delivered, potentially affecting stress and leakage outcomes. Furthermore, the cavity dimensions, in line with minimally invasive dentistry principles, were atypical, deliberately chosen to enhance the observation of materials’ marginal adaptation properties. The 1000 cycles aging procedure might not be able to replicate clinical conditions. Future analyses should concentrate on the marginal adaptation following thermomechanical aging. The silver nitrate penetration method may have limitations, in many in-vitro leakage studies [53], it has been noted that the tracers used are actually smaller than bacteria, food, or fluids encountered by restorations in the oral environment. Consequently, the observed leakage amounts may not accurately reflect real oral conditions. Also, there can be areas where the tracer does not penetrate, yet the interface between the restoration and tooth indicates a failure of adaptation. This situation could potentially lead to a misinterpretation of the results.

The influence of brand-specific properties, as evidenced in our study, indicates a more complex interaction between material composition and performance than previously understood. This observation is particularly relevant in the context of the diverse array of dental composites available in the market, each with its unique formulation. Findings of this study, therefore, contribute to a more tailored approach in selecting dental composites, emphasizing the need for dentists to consider the specific properties of each material.

## Conclusion

This research highlights the significant role of chemical composition in the marginal adaptation of bulk-fill resin composites in Class II restorations, challenging the prevalent notion that material viscosity is a key determinant. X-tra groups (XB and XF) showed the highest leakage values for bulk fill composites in both different viscosity groups. Bulk-fill composites could be viable alternatives to conventional composite resin in terms of marginal adaptation.

## Data Availability

No datasets were generated or analysed during the current study.
